# Nano-Strategies for Enhancing the Bioavailability of Tea Polyphenols: Preparation, Applications, and Challenges

**DOI:** 10.3390/foods11030387

**Published:** 2022-01-29

**Authors:** Li Niu, Ziqiang Li, Wei Fan, Xiaohong Zhong, Miao Peng, Zhonghua Liu

**Affiliations:** 1 Key Laboratory of Tea Science of Ministry of Education, Hunan Agricultural University, Changsha 410128, China; nl260122288@163.com; 2 National Research Center of Engineering and Technology for Utilization of Botanical Functional Ingredients, Hunan Agricultural University, Changsha 410128, China; 3 College of Horticulture, Hunan Agricultural University, Changsha 410128, China; dx777@hunau.edu.cn (Z.L.); xh-zhong@163.com (X.Z.); 4 College of Food Science and Technology, Hunan Agricultural University, Changsha 410128, China; fanwei99098184@163.com

**Keywords:** tea polyphenols, epigallocatechin gallate, catechin, nanoparticles, nanoemulsions, nanomicelles, nanolipids

## Abstract

Tea polyphenols (TPs) are among the most abundant functional compounds in tea. They exhibit strong antioxidant, anti-inflammatory, and anti-cancer effects. However, their instability and low bioavailability limits their applications. Nanotechnology, which involves the use of nanoscale substances (sizes ranging from 1 to 100 nm) to improve the properties of substances, provides a solution for enhancing the stability and bioavailability of TPs. We reviewed the preparation, performance, effects, and applications of different types of TPs nanocarriers. First, we introduced the preparation of different nanocarriers, including nanoparticles, nanoemulsions, nanomicelles, and nanolipids. Then, we discussed various applications of tea polyphenol-loaded nanocarriers in functional ingredient delivery, food quality improvement, and active food packaging. Finally, the challenges and future development directions of TPs nanocarriers were elucidated. In conclusion, a nano-strategy may be the “key” to break the application barriers of TPs. Therefore, the use of nano-strategies for the safe, stable, and efficient release of TPs is the direction of future research.

## 1. Introduction

The various natural compounds in plants are a treasure trove given to mankind by nature, and mankind has a long history of using plant compounds to regulate various health problems [1,2]. Polyphenols are the most consumed natural antioxidants in the daily diet. Their total daily intake by the human body is about 10 times the intake of vitamin C, and about 100 times the intake of vitamin E and carotenoids [3]. As natural dietary factors, polyphenols have various regulatory functions on human health [4]. Tea polyphenols (TPs) are among the many plant polyphenols. They are mainly derived from tea plants and account for about 20–30% of the dry weight of tea. Apart from being the main functional components of tea, TPs determine the color, aroma, taste and other quality indicators of tea [5]. Among TPs, catechins have the highest concentrations, accounting for about 60–80% of total TP weights. These catechins include four main catechin monomers (Epicatechin, EC; Epigallocatechin, EGC; Epicatechin gallate, ECG, Epigallocatechin gallate, EGCG) and other catechin oxidation polymerization products (Theaflavins, thearubicins, and theafucins among others) formed during processing [6,7].

Tea, which has a unique taste and health benefits, is one of the three largest non-alcoholic beverages in the world. With the development of new functional drinks, tea and TPs are increasingly being used as beverage raw materials and natural additives, which greatly increases the chance of the human body ingesting TPs from the diet. Tea, as a natural plant rich in tea polyphenols, is a rich and low-cost raw material for TPs. Macroporous resin separation, recrystallization, membrane separation, and other technologies have been widely used in industrial production of tea polyphenols. Industrial tea polyphenols with a purity of more than 95% can be obtained at a low cost [8,9,10]. In the year 2020, the total tea polyphenol output in China was about 5000 tons, which were mainly used as raw materials or food additives in food processing, healthcare products, and daily chemical products. Biologically, TPs have powerful antioxidant and anti-inflammatory effects [11], important regulatory roles in human metabolic syndromes [12] and cardiovascular diseases [13], resistance to malignant tumors [14], nerve protection [15], and delayed aging [16]. Therefore, TPs are widely applied in the fields of biomedicine, dietary nutrition, and food daily chemicals [5]. Due to the wide range of consumer groups, a low-cost material base, and multiple health benefits, TPs are some of the most promising substances in plant polyphenols.

As we all know, most of the polyphenols have the defects of low bioavailability, low absorption efficiency in the body, and instability *in vitro*, which greatly limits the application of many polyphenols, including TPs [17,18]. Nanotechnology has several applications in the food field, including in food safety and quality, food packaging and sensory improvement, the targeted delivery of compounds, as well as in the improvement of bioavailability [19,20]. The use of food protein particles and chitosan as nanocarriers for plant polyphenols can significantly promote the intestinal absorption of phenolic compounds [21]. Furthermore, polysaccharide-bioactive polypeptide nanoparticles can also be used as valuable nanocarriers for encapsulating small-molecule polyphenols to provide these functional polyphenols with better bioavailability [22]. Nanoencapsulation packaged phenolic substances, including curcumin and carvacrol, play an active role in food processing and packaging, which can ensure the color as well as the quality of polyphenols and improve the performance of packaging materials [23,24]. Even though some nanoparticles can play various roles after being loaded with polyphenols, they are often restricted in the actual food processing process. For instance, the solubility of chitosan is affected by pH [25], while elevated levels of TiO_2_ (Titanium Dioxide) particles negatively impact the bioavailability and biological activity of polyphenols in food [26].

The development of nano-TPs has undergone self-nanoization and hybridization of TPs with various nanomaterials. Currently, nano-sized carriers for TPs include nanoparticles (such as polymer nanoparticles), nanoemulsions, nanomicelles, and nanoliposomes [27,28]. These nano-strategies have greatly improved the utilization of TPs and provided a material basis for the precise delivery of TPs, the development of functional foods, as well as applications of new food packaging materials [29]. The loading of TPs into nano- and micro-systems greatly improves their delivery and bioavailability in vivo, which is associated with the fact that nano- and micro-systems can stabilize the structure and activity of TPs in unfavorable environments, such as the gastrointestinal tract [30]. Based on the different biophysical properties and biological functions of tea polyphenols, the loading and delivery methods of nanoparticles are also different. For instance, encapsulating EGCG, the main monomer of TPs, in a nano-scale double-walled carrier composed of chitosan and β-lactoglobulin can effectively control the release of EGCG in the gastrointestinal tract and improve its antioxidant activities and its availability in the body [31]. The binding methods of tea polyphenols and nanocarriers are diverse, and they include ultrasound and electro-fluidic encapsulation, among others. These binding methods affect the properties of tea polyphenol nanoparticles [32]. These studies show the significance of TP nanocarriers of different nanometerizations in improving the functions of TPs.

Apart from their biological activities, tea polyphenols have huge application potentials in food nutrition, food processing, and packaging. However, their applications are limited by low bioavailability [33], low in vivo absorption rate, and low *in vitro* instability. Green functionalized nano-TPs, which have been developed based on nano-strategies, have the ability to improve the performance defects of TPs. In addition, there is a need to regulate the safety of nanocarriers and their applications in the food field. In 2011 and 2014, the Food and Drug Administration (FDA) issued guidelines on the regulation and definition of products involving nanotechnology, which preliminarily defined the use of nanotechnology and materials in the food field (including dietary supplements and nutrient delivery among others) [34,35]. All wall materials, stabilizers, and natural and synthetic additives used in food nanomaterials must be non-toxic and recognized as safe by the FDA. This provides a technical guide for the application of nano-TPs in food.

**Figure 1:**
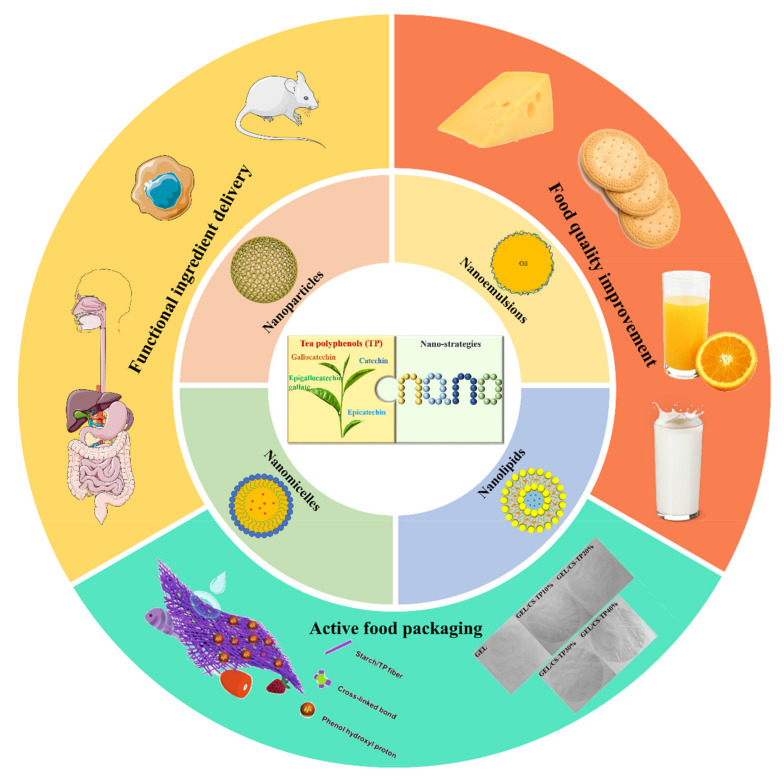
Bioavailability and application prospects of tea polyphenols improved by nano-strategies. (The figures of active food packaging are cited from [36,37], Copyright © 2021, Elsevier; Copyright © 2020, Elsevier).

We reviewed the preparation technology of functionalized nano-TPS and their applications in targeted nutrition delivery, functional food development, and food packaging (Figure 1). On this basis, future expectations and challenges of functional nano-TPs are elucidated, which may inform the application and development of nano-TPs.

## 2. Preparation Methods of TPs Nanocarriers

**Figure 2:**
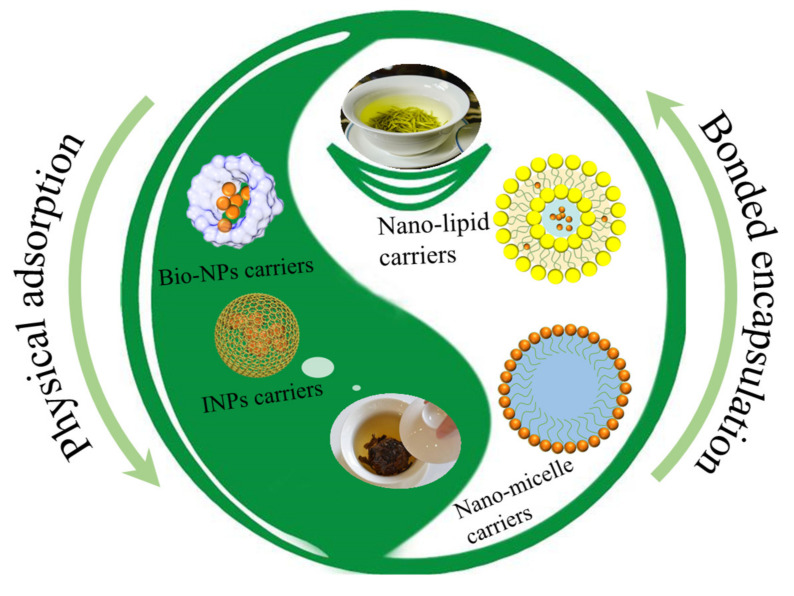
Schematic presentation of the nano-encapsulation of tea polyphenols by physical adsorption and chemical bonding.

Nano-technology is a new high-tech discipline that involves the manipulation of atoms and molecules in the nanometer scale to process materials, manufacture products with specific functions, or study a substance to master the laws of motion and properties of its atoms and molecules. Typical tea polyphenols have a low oral bioavailability and photothermal instability [38,39]. Despite the reported efficacies of active compounds in tea against a number of diseases *in vitro*, the above limitations pose a serious challenge for their in vivo applications. Recently, nanotechnology has been introduced in research on TPs. This is attributed to the physical and chemical properties of nano-materials, including surface and interface effects, small size effects, and quantum effects, which are not available in traditional materials [40]. Studies on the improvement of the bioavailability of TPs through nanoscience and nanotechnology include nano-particles, nano-emulsions, nano-micelles, nano-liposomes, and other nano-materials [41]. The stability and bioavailability of TPs can be improved through nanomaterial encapsulation (physical adsorption and chemical bonding, Figure 2).

### 2.1. Nano-Particles

Nano-particles are solid colloidal particles that range in size from 10 to 1000 nm with a large external surface area, a high hole ratio and surface energy, and many surface-exposed atoms. Due to their earlier mentioned properties, including surface effects, small size effects, and macroscopic quantum tunneling effects, nano-particles have been used to improve the bioavailability of TPs in vivo and *in vitro*, enhancing their stability in the physiological environment and achieving sustainable release [42]. Several types of nano-particles have been developed for use in improving the bioavailability of TPs [43].

**Figure 3:**
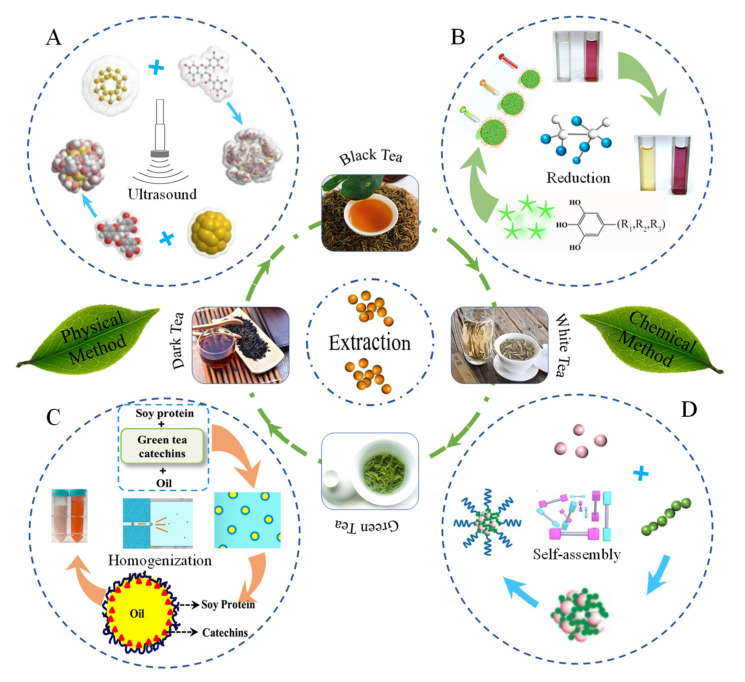
Schematic presentation of nano-encapsulated TPs by ultrasound (**A**), reproduced with permission [44], Copyright © 2011, Elsevier; reduction (**B**), reproduced with permission [47,50], Copyright © 2015, Elsevier; Copyright © 2021, Elsevier; homogenization (**C**), reproduced with permission [51], Copyright © 2018, Elsevier; and self-assembly methods (**D**), reproduced with permission [52], Copyright © 2014, Nature Publishing Group.

Due to their uniform pore channels, easy functionalization, biocompatibility, high specific surface area, large pore capacity, and biodegradability, inorganic nano-particles (INPs) have been widely used for drug encapsulation and delivery. Specifically, gold nano-particles are used as carriers, which can effectively protect and enhance the bioavailability of tea active compounds. Active compounds in tea are usually loaded in gold nanoparticles through physical adsorption. Through physical adsorption with the help of ultrasound or apex vibration, Hsieh et al. [44] prepared EGCG-nanoparticles by compounding EGCG and gold nanoparticles (Figure 3A). The optimal combination of EGCG and gold nanoparticles was determined by assessing the physicochemical properties of the EGCG–gold nanoparticle complex. Moreover, the characterization of the EGCG–gold nanoparticle complex was performed by high-performance liquid chromatography (HPLC) to determine EGCG encapsulation. As a result of the distribution of gold nanoparticles on the surface of the composite, the zeta potential of EGCG–gold nanoparticles had increased to +21.0 ± 5.0 mV. Meanwhile, SEM images confirmed the complexation between EGCG and gold nanoparticles [44]. In their subsequent study, Hsieh et al. [45] investigated the experimental details of optimization. Gold nanoparticles were characterized by photon correlation spectroscopy (PCS), transmission electron microscopy (TEM), and dynamic light scattering (DLS) technologies. It was found that EGCGs were attached to the gold nanoparticles through physical adsorption, and the sizes of freshly prepared EGCG–gold particles were about 500 nm with a zeta potential of 21 mV. Due to electrostatic repulsion, EGCG–gold nanoparticles are not susceptible to agglomeration [45]. As a high-efficiency physical field, ultrasound, which has a unique ability to promote dissolution, has been widely applied in biomedicine and in the food industry. Through ultrasound for physical adsorption, Yuan et al. [46] developed EGCG–gold nanoparticles composed of EGCG and gold nanoparticles. Under ultrasonic conditions, the loading capacity of gold nanoparticles for EGCG increased. Ultrasound plays a vital role in the loading of TPs, including promoting EGCG dissolution, accelerating the movement of active molecules of TPs, and strengthening physical adsorption, among others. The reduction method has also been used to prepare conjugated EGCG and gold nanoparticle complexes [46]. EGCG nanoparticle conjugates obtained via the in situ reduction method have attracted attention from researchers. Using the chloroauric acid reduction method, Mukherjee et al. [47] prepared purple-red spherical EGCG–gold nanoparticle conjugates with diameters between 24 and 30 nm (Figure 3B). During the synthesis of the conjugates, the gold nanoparticles directly interacted with the TPs. The synthesized EGCG–gold nanoparticles exhibited excellent stability at 4 °C for 6 months. Mixing the TPs with gold nanoparticles enhanced their stability and bioaccessibility. Silver nanoparticles have also been shown to exhibit comparable abilities [47]. Applications of silver nanoparticles in enhancing the stability and bioavailability of TPs can be realized through simple physical encapsulation. In terms of encapsulation efficiency, silver nanoparticles have a large specific surface area and can adsorb more TPs compared to gold nanoparticles. Furthermore, adsorption can be specifically enhanced under the actions of ultrasound or microwaves. Another potential strategy for improving the stability and bioavailability of TPs involves the applications of TPs as guest reducing agents and stabilizers in reactions with host nanocarriers. The involvement of white tea extract as a capping agent in the synthesis of silver nanoparticles was revealed through various spectroscopic techniques, including UV–Vis spectra, X-ray diffraction (XRD), and Fourier-transform infrared spectra (FTIR Spectra) analysis. TEM images of mixed nanoparticles showed that the combination of tea extract and silver nanoparticles resulted in spherical and pseudo-spherical shapes as well as smooth surfaces [48]. To assess the toxicity of TPs and silver nanoparticle complexes, Barbasz et al. compounded EGCG with silver nanoparticles, and found that EGCG nanoparticles disrupted the integrity of cell membranes and penetrated into cells to cause DNA damage, resulting in reduced mitochondrial activities [49]. However, the significance of single gold or silver nanoparticles is limited to improving the encapsulation efficiency of TPs. Meanwhile, silver nanoparticles that are used to enhance the stability and bioaccessibility of TPs are associated with biotoxicity.

**Figure 4:**
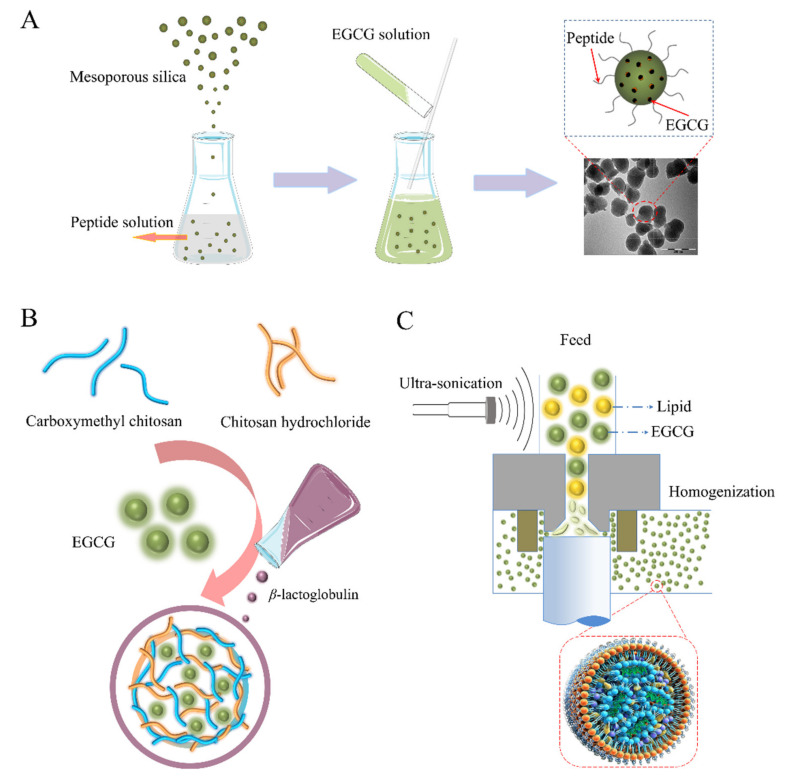
(**A**) The loading of EGCG into nanoparticle carriers functionalized with the shell-core structure. Reproduced with permission [54], Copyright © 2015, American Chemical Society. (**B**) Schematic illustration of the encapsulation of EGCG by PNPs with double-wall structure. (**C**) Nano-lipids prepared by sonication and homogenization for enhanced stability and bioavailability of EGCG.

Recently, green synthesized mesoporous silica nanoparticles with unique properties, including tunable particle sizes and morphology; tailored mesoporous structures; uniform, tunable pore sizes; high chemical and mechanical stability; high surface area and pore volume; as well as high drug loading capacity and convenient surface functionalization have been used for complexation with TPs [53]. The loading of EGCG into the mesoporous silica nanoparticle system functionalized with the shell-core structure (Figure 4A) by mesoporous physisorption and surface electrostatic attraction confirmed that biological activities of EGCG can be protected in the nanoparticle system [54]. In nano-hydroxyapatite/mesoporous silica systems, EGCG has shown excellent potential, including in dentin surface occlusion and biofilm generation to inhibit daily tooth erosion and wear. Furthermore, epigallocatechin-3-gallate encapsulation in nanohydroxyapatite/mesoporous silica nanoparticles was achieved via a homogeneous precipitation technique. A large number of EGCG molecules was adsorbed into internal pores of the mesoporous silica, as revealed by TEM images. The effective loading rate of EGCG was determined to be 11.29%. The feasibility of the obtained EGCG–mesoporous silica for the treatment and restoration of dentin surfaces was evaluated by field emission scanning electron microscopy (SEM) and dentin permeability measurements. Laser confocal microscopy (LCM) showed that the composite nanomaterials were able to inhibit the proliferation of *Streptococcus* mutans [55]. Then, the composition and release characteristics of the mesoporous silica-based EGCG nano-hydroxyapatite delivery system was investigated through adsorption and precipitation. It was found that EGCG molecules occupied the internal pore channels of the mesoporous silica, resulting in the reduced specific surface area and porosity of the silica. In addition, the mesoporous silica delivery system had the potential to continuously release EGCG and provided an effective biological barrier to protect the exposed dentin in the oral cavity [56].

Studies on precision medicine and personalized nutrition demand strategies have shown the need for specific nanoparticles, including light, thermal, magnetic, and pH-responsive materials for specific needs. The surface modification of silica nanoparticles can lead to more functionalities, such as photothermal and pH responses. Li et al. [57] evaluated the electronegativity property of EGCG polyhydroxyl groups and loaded them into amidated mesoporous silica nanoparticles through electrostatic interactions. This revealed a photothermal therapeutic agent with high drug loading efficiency and a good pH-dependent drug release, which is a potential therapeutic option for tumors [57]. Iron with magnetic properties is also a substrate for building nanoparticles. For instance, iron-polyphenol nanoparticles are emerging phototherapeutic agents that are usually prepared with non-toxic plant polyphenols as the main components. Through the hydrothermal method, Qin et al. [50] combined different polyphenols (such as epigallocatechin gallate, gallic acid, and epicatechin as ligands) with low toxicity iron and selected optimal iron contents to make phototherapeutic agents for cancer (Figure 2B). The established polyphenol–iron nanoparticles were uniformly dispersed in water, exhibited a higher absorption/scattering ratio, and could therefore convert more light energy into heat energy. In addition, the nanoparticles were effective at inhibiting in vivo tumor growth [50]. Through an oil–water biphase stratification coating strategy, Yan et al. [58] developed a composite magnetic material of tea polyphenols and nanoparticles with the ability for amyloid inhibition, drug carriage, and facile separation. By applying the imprinting technology with the help of ultrasound, EGCG was loaded into nanoparticles with Fe3O4 as the shell and silicon dioxide as the core. The loading rate was about 52 μg mg^−1^ [58]. Although the silica-based TPs delivery system enhances the loading of TPs, its in vivo biocompatibility is not satisfactory. Recently, an edible metal organic framework (MOF), an emerging porous nanomaterial, has been used to enhance the stability and bioavailability of TPs. The encapsulation of polyphenols into the cyclodextrin-based metal organic framework (CD-MOF) through physical adsorption has been reported by Ke et al. [59]. The loading capacity of CD-MOF for EGCG was controlled by EGCG concentrations. When the loading rate was about 20 μg mg^−1^, the EGCG encapsulated in CD-MOF exhibited a similar efficacy as free EGCG [60]. However, the single MOF materials cannot overcome the low loading rate and controlled release limitations of TPs [61]. Therefore, the combined structure of MOF core and silica shell prepared for loading catechins have the potential for overcoming this limitation. Encapsulation of catechin in MOF and silica was associated with a high loading capacity and antioxidant activities [59]. However, due to their safety and health concerns (including metabolism-associated accumulation and their ability to cross the blood–brain barrier), INPs are not commonly used in edible products [32].

In addition to INPs, biopolymer-based nanoparticles (PNPs) have a stable morphological structure. PNPs can be designed, synthesized, and prepared at the molecular level by selecting the appropriate polymerization methods and monomers. The size and particle uniformity of polymer nanoparticles are easy to control and have the advantages of small size effects, surface size effects, and biocompatibility. Currently, PNPs are widely used in TP delivery. The ion gel method has been used to prepare nanoparticles with improved encapsulation efficiencies and stability (Figure 4B). To prepare spherical starch PNPs, Liu et al. used the ionic gelation method to electrostatically interact with negatively charged carboxymethyl debranched starch and positively cationized debranched starch [62]. Epigallocatechin gallate (EGCG) was successfully incorporated into PNPs, and the maximum encapsulation rate was 84.4% [62]. Similarly, through the ionic gelation approach, Liang et al. prepared chitosan/β-lactoglobulin PNPs with a core-shell double-walled structure as EGCG nanocarriers [25]. During preparation, the carboxymethyl chitosan solution was added dropwise to the chitosan hydrochloride containing EGCG to form a primary coating through ion complexation. Then, β-lactoglobulin was added to pass through the ionic gelation, after which a second coating was formed [25]. FT-IR spectroscopy revealed that the PNPs had combined with EGCG via hydrogen bonds to achieve EGCG encapsulation [25]. With regards to the preparation of protein nanoparticles, the self-assembly method, which is a method that does not require organic solvents, is a good choice. Gou et al. used the self-assembly of EGCG and ovalbumin under high temperature conditions to prepare ovalbumin PNPs encapsulating EGCG [63]. EGCG achieves a high encapsulation efficiency through two ways: the adsorption to EGCG binding sites in ovalbumin molecules and physical encapsulation with ovalbumin molecules [63].

### 2.2. Nano-Emulsions

Nano-emulsions with droplet diameters in the range of 20 to 200 nm are thermodynamically unstable heterogeneous systems. They are composed of dispersions of nanoscale droplets formed via shear-induced rupture. When the dispersal phase is oil, while the continuous phase is water, the resulting emulsion is referred to as an oil-in-water (O/W) nano-emulsion. However, when the water phase is dispersed in the oil phase, the resultant emulsion is referred to as a water-in-oil (W/O) nano-emulsion.

The preparation of appropriate nano-emulsion delivery systems to improve the stability and bioavailability of TPs decreased damage to orally administered polyphenols and enabled a sustainable as well as responsive release of TPs. Through high pressure homogenization, Peng et al. [51] prepared O/W nano-emulsions with particle sizes of 99.42 ± 1.25 nm by combining TPs, corn oil, and polysorbate 80 (Figure 3C). Furthermore, in the final nano-emulsion, the loading capacity of TPs was 4 mg/mL. The resultant nano-emulsion exhibited a high stability when stored under different temperatures for 20 days. *In vitro* simulated digestion data revealed that EGCG bioavailability in the nano-emulsions had increased, when compared to aqueous solutions. However, the bioavailabilities of EGC, EC, and GCG were markedly reduced. In addition, the plasma concentration–time profile in rats revealed a controlled release and increased bioavailability of EGCG using nano-emulsions [51]. Through high-pressure homogenization, Bhushani et al. [64] prepared a green tea catechins nano-emulsion using soy protein, sunflower oil, and catechins (Figure 3C). At a storage temperature of 4 °C, the emulsion droplet size, pH, and catechin concentrations in the catechin nano-emulsion were revealed to be stable. The bioaccessibility of the nano-emulsified form of catechin increased by 2.78-fold compared to the unencapsulated form. In addition, the intestinal permeability for catechins was significantly increased, as revealed by the Caco-2 cell model. The soy-protein-based nano-emulsion improved the stability, bioaccessibility, and permeability of green tea catechins [64]. To improve EGCG stability and reduce its associated side effects in human lung cancer treatment, through ultrasonic dispersion, Chen et al. [65] used EGCG, lecithin, and Tween 80 to prepare transparent EGCG nano-emulsions with a yellow appearance. During the 120 day storage period, there were no significant changes in particle sizes, the polydispersity index (PDI), the zeta potential, and the encapsulation efficiency of the EGCG nano-emulsion. The assessment of the *in vitro* bioavailability of TPs revealed that the EGCG nano-emulsion inhibited H1299 lung cancer cell proliferation and effectively suppressed colony formation, as well as migration. This indicates that the nano-emulsions are a potential strategy for improving the bioavailability of TPs [65]. In the presence of ultrasonic waves, nano-emulsions can be complexed with TPs from various sources. Tea leaf wastes (by-products obtained during the processing of tea beverages) contain large amounts of catechins, which are often treated as wastes and, therefore, ignored during the actual production process. Tsai and Chen (2016) [66] prepared catechin nano-emulsions from tea wastes through the ultrasound approach. They found that the drop size distribution of the TPs nano-emulsion was in the range of 10 to 13 nm (confirmed by DLS and TEM). In addition, over a storage period of 120 days at 4 °C, the TPs nano-emulsion exhibited a high stability, which was attributed to high electrostatic repulsion forces between the droplets. The encapsulation efficiency of TPs in the nano-emulsion was 88.1% [66]. Furthermore, the catechins released from the nano-emulsion induced the apoptosis of PC-3 cells, indicating that the biological activities of TPs had effectively been improved [66]. Encapsulation of catechins from oolong tea wastes in a nano-emulsion prepared from lecithin, Tween 80, and water by stirring under the action of ultrasound was also reported by Lin et al. [67]. The resulting nano-emulsion had a particle size of 11.3 nm, a zeta potential of—67.2 mV, an encapsulation rate of 83.4% for catechins, and a high emulsion stability, implying better effects in the inhibition of DU-145 cell proliferation, relative to free catechins [67]. Nano-emulsions have been shown to improve the stability and bioavailability of TPs, while maintaining their loading rates in carriers. In conclusion, nano-emulsions may be used as a basis for protection and enhancement of the bioavailability for TPs.

### 2.3. Nano-Micelles

Nano-micelles are amphiphilic colloids that regulate the diameter of micelles to between 20 and 200 nm, which is on the basis of traditional micelles [68]. Micelles are spherical particles with a hydrophobic core and composed of surfactants, resulting in drug encapsulation in the hydrophobic core or converse hydrophilic core [69]. Micelles can enhance the solubility of TPs in water, prolong its blood circulation time, increase its specificity when targeting diseased tissues by enhancing penetration and retention, as well as reduce the toxicity of TPs to body organs [70].

The formation of micelles occurs through two forces. The first one involves the attraction that leads to molecular binding, while the other involves repulsion, which prevents the indefinite growth of micelles to distinct macroscopic phases. The sizes of micelles are determined by hydrophobic forces that enclose the hydrophobic chain in the core and the repulsive force for repulsive volume between the chains that limits its size. Chung et al. [52] prepared a self-assembled micellar nanocomposite that is composed of green tea catechins and proteins (Figure 3D). The oligomerized EGCG was used to stabilize the micellar core by enhancing the binding property of EGCG with the herceptin protein, while polyethylene glycol was used to form the outer shell through Baeyer reactions between an aldehyde group and the nucleophilic A ring of EGCG. Nano-micelles that are formed via sequential self-assembly of EGCG derivatives and anticancer proteins can maintain their integrity and good stability at 37 °C for 15 days. In addition, after 1000-fold dilutions, there were no size reductions, implying excellent stability as a function of suspension dilution for good anti-cancer effects [52]. Because of its safety, bioavailability, and stability, the casein-based micellar is a natural carrier that has been applied in different nutraceuticals, such as polyphenols [71]. In a previous study, EGCG-loaded casein molecules were transformed into re-combined casein micelles (r-CMs) by induction effects of hydrated salts, which were effective at protecting EGCG against heat-induced degradation. The encapsulation efficiency of r-CMs was 85%, while the average particle size was 66.2 nm [72]. In vivo, EGCGs encapsulated in nano-micelles have been shown to exhibit excellent colloidal stability and biological activity. Sánchez-Giraldo et al. prepared a mixed micelle to encapsulate EGCG in situ [73]. Briefly, first, EGCG was loaded into the earlier prepared block copolymer and sodium dodecyl sulfate blend micelles. Then, it was subsequently covered with chitosan on the micelle surface. The average radius of the mixed micelle was 35 nm, whereas the encapsulation rate of EGCG in the micelle was 83%. This delivery maintained the in vivo antioxidant activities of EGCG. As natural cationic polymers, chitosan micelles have been shown to be safe for drug delivery [74]. In a previous study, chitosan–catechin conjugates were prepared by dispersing catechins in a Tris-HCl buffer containing chitosan by ultrasound. Chanphai and Tajmir-Riahi [75] found that the coupling of catechin and chitosan occurred via hydrophilic, hydrophobic, and hydrogen bonds. The resulting chitosan micelles effectively protected catechin and improved its bioavailability [75]. In addition, chitosan can be used to construct polymer micelles. Ding et al. [76] prepared a simple self-assembled nano-micelle composed of protamine and small interfering RNA (siRNA) in Hank’s Balanced Salt Solution for encapsulating EGCG. The polyhydroxy EGCG was absorbed and loaded on siRNA/protamine micelle via non-covalent interactions with siRNA. The EGCG-loaded micelle exhibited excellent selectivity and tumor growth inhibition effects in xenograft MDA-MB-231 tumor-bearing mice with little toxicity to normal tissues and organs [76].

Therefore, polymer nano-micelles are potential strategies for enhancing the stability and bioavailability of TPs, which is attributed to its strong loading capacity caused by the excellent cavity structure, high safety, low toxicity, and great biocompatibility.

### 2.4. Nano-Lipids

Nano-lipids are hollow structures with particle sizes of less than 1000 nm. Their bilayer structures are similar to those of skin cell membranes. Compared to the conventional biomaterials for delivery systems, nano-lipids are associated with various advantages, including increased solubility, enhanced stability, enriched permeability, and bioavailability [77].

Due to their lipid cores or hydrophobic surfaces, applications of lipids-based materials, including liposomes, solid lipid nanoparticles (SLNs), and nanostructure lipid carriers (NLCs), are advantageous for enhancing the bioavailability and therapeutic efficacy of TPs. Given that they have the ability to improve drug pharmacokinetics, some lipid-based nanoparticles with particle diameters ranging from 80 to 300 nm have been developed as drug carriers. The limitations associated with the low stability and bioavailability of TPs in vivo will potentially be addressed by lipid nanoparticles (Figure 4C). For instance, through coupled high-shear homogenization and ultrasonication techniques, Frias et al. developed a method for preparing SLNs and NLCs [27]. The obtained SLNs and NLCs liposomes had an average hydrodynamic diameter of between 300 and 400 nanometers, implying a narrow size distribution. Granja et al. (2017) [78] prepared a nanostructured lipid using high-shear homogenization and ultrasound technology. Then, they evaluated its encapsulation efficiency for EGCG, oral absorption, and bioavailability. The encapsulation rate was 85%, the EGCG-loaded lipid nanoparticles achieved a controlled release of EGCG, while the storage stability was up to 8 weeks [78]. In addition, cationic nano-liposomes can be obtained from multi-layer emulsion templates via ultrasound. Fangueiro et al. [79] used a double-emulsion technique to prepare a cationic EGCG-loaded nano-lipids. These EGCGs were encapsulated in situ inside liposomes, resulting in improved stability and the prolonged or controlled release of EGCG by the obtained nano-lipid [79]. The combination of ultrasound and phase invasion technology can also be used to prepare nanoliposomes. Barras et al. [80] used this technique to prepare nanoliposomes loaded with 95% EGCG. The EGCG-loaded liposomes maintained their stability for 10 weeks [80]. A versatile high shear homogenization method can also be used to prepare nano-liposomes. Manea et al. [81] evaluated the feasibility of the modified high shear homogenization method for the co-encapsulation of green tea extracts with lipids. The prepared TP-loaded nano-lipids exhibited excellent physical stabilities and enhanced antioxidant activities relative to free TPs [81].

Nano-lipid materials have the ability to improve the stability and bioavailability of TPs. Furthermore, it is postulated that in the future, nano technologies for enhancing the stability and bioavailability of TPs, such as electro-spinning and electro-spraying, will be developed.

## 3. Applications of TPs Nanostrategies in the Food and Nutrition Sector

### 3.1. Functional Ingredient Delivery

Currently, nano-carriers including nanoparticles, nanoemulsions, and nanolipids, among others, have been widely used as a good strategy to solve the challenge of the stability of TPs by enhancing their efficient delivery, release, and absorption in the human body (Table 1).

Various enzymes in the gastrointestinal tract as well as the low pH environment reduces the oral effectiveness of TPs. Various nano-carriers have been used to achieve the sustainable release of TPs in the human gastrointestinal tract, thereby improving its stability and bioavailability. Nanolipids are prepared from physiological lipids; therefore, they are safe, stable carriers to ensure the controlled release of TPs in vivo. Granja et al. prepared a folic acid-functionalized EGCG nanostructured lipid carrier to achieve stable release of EGCG in the gastrointestinal tract [78]. The folic acid acts as a ligand to target intestinal epithelial cells expressing folate receptors, thereby enhancing the release of EGCG from the intestines [78]. Solid lipid nanoparticles made from a mixture of cocoa butter and food-grade surfactants have been used for the encapsulation and delivery of EGCG. Under intestinal conditions at pH 6.8, these nanoparticles can improve the stability of EGCG [85]. Polysaccharides such as chitosan and starch, which can be obtained from various sources, are associated with good biocompatibility and non-toxic effects. Proteins are prone to structural and conformational changes under different conditions. Therefore, polysaccharides and proteins are usually used as raw materials to prepare carrier nanoparticles with good delivery and controlled release of TPs. Liang et al. developed a chitosan/β-lactoglobulin nanoparticle, which achieved an extended release of oral EGCG in the gastrointestinal tract [25]. Pepsin resistance of the β-lactoglobulin in the outer layer of the nanoparticle allowed EGCG to be released slowly in simulated gastric conditions, while β-lactoglobulin digestion on the outer layer of the nanoparticle led to the rapid release of EGCG in simulated small intestine conditions [25]. After the degradation of the outer wall of β-lactoglobulin, the exposed chitosan chains can adhere to the intestinal wall (attributed to adhesion properties of chitosan to the gastrointestinal mucus), thereby increasing the residence time of nanoparticles in the intestinal tract, which improves the bioavailability of EGCG [25]. Similarly, Gao et al. prepared β-lactoglobulin/gum arabic composite nanoparticles whose encapsulation with EGCG ensured their continuous release in a simulated gastrointestinal tract [82]. Furthermore, the sensory evaluation of EGCG-encapsulated β-lactoglobulin nanoparticles revealed that the bitterness (general bitterness and lagging bitterness) as well as the astringency of the nanoencapsulated EGCG were lower than those of the unencapsulated EGCG at the same concentrations, indicating that β-lactoglobulin nanoparticle encapsulation is beneficial in improving the sensory effects of EGCG [86]. In addition to β-lactoglobulin, other protein-based nanoparticles are also used to enhance the release stability of TPs. Yang et al. prepared phytoferritin–chitosan–epigallocatechin nanoparticles through a one-step heat treatment method at 55 °C. The protective effects of ferritin cages and chitosan improved the stability of epigallocatechin in the gastrointestinal tract [83]. Starch nanoparticles have also been used to achieve the controlled release of EGCG in the gastrointestinal tract. Liu et al. used the ionic gelation method to prepare debranched starch nanoparticles, which achieved the controlled release of EGCG in simulated gastric juice and intestinal juice environments [62].

Apart from nanoparticles, nanoemulsions are ideal carriers for TPs. These droplets, with sizes smaller than 100 nm, are dynamically stable [87]. In the *in vitro* simulated digestion experiment of Peng et al., the oil-in-water nanoemulsion prepared by high-pressure homogeneous emulsification with corn oil and polysorbate-80 effectively improved the oral bioavailability of EGCG [51]. Similarly, Gadkari et al. used the high-pressure homogenization technology to prepare an oil-in-water nanoemulsion with sunflower oil as the lipid carrier and Tween 80 as the emulsifier to achieve the sustained release of catechins in simulated gastric conditions [84].

The applications of nanogels in improving the delivery and bioavailability of TPs have not been conclusively evaluated. However, nanogels, as a three-dimensional polymer chain networks, are good potential TPs carriers. Piran et al. reported that the encapsulation of green tea extracts in chitosan–citric acid nanogels improved their antioxidant activities [88], providing a reference for future applications of nanogels to deliver TPs.

### 3.2. Food Quality Improvement

In addition to the earlier-mentioned use of tea polyphenol nanocarriers to achieve a targeted release of TPs in different body parts, they can also be used to improve food quality or realize food functionalization. Tea polyphenol nanocarriers have been used in various foods, including dairy products, beverages, and baked products, among others (Table 2).

It has been confirmed that encapsulating catechins and EGCG in nano-liposomes and adding them to cheese can not only improve the stability of TPs during cheese storage but also increase the antioxidant capacity and total phenolic content of cheese [89,90,94]. Moreover, the supplementation of green tea catechin nanocapsules to hard low-fat cheese may not affect the composition and pH of cheese [89]. In addition, Rashidinejad et al. performed *in vitro* simulated gastrointestinal digestion experiments to show that the TPs in full-fat hard cheese can still be recovered from the gastrointestinal digesta of cheese samples after 6 h, implying that nano-liposomes can effectively protect TPs in cheese from degradation [90]. 

Apart from cheese, a catechin nanoemulsion prepared by ultrasound was used in high-pressure processed coconut milk to enhance its antioxidant activities and prolong its shelf life. Furthermore, the high-pressure processing had no effects on the physical stability and antioxidant activities of the catechin nanoemulsion [91]. Nanocarriers containing TPs can also be used in other liquid drinks. Feng et al. prepared low-methoxy pectin-coated nanoliposomes encapsulated with resveratrol and epigallocatechin and added to orange juice [92]. The low-methoxy pectin of the nano liposomes could be bridged with metal ions in orange juice to form a network gel, so that the nanosomes could maintain good stability after pasteurization and have stronger antioxidant activity [92]. 

Bakery food is common, and the feasibility of adding nanocarriers containing TPs to improve bakery food functionality is being evaluated. Through the electrospray technique, Gómez-Mascaraque et al. developed protein microcapsules containing green tea extracts and applied then in biscuit dough. Even though the microcapsules had good heat resistance, they did not improve the antioxidant capacities of the biscuit dough [93]. In addition, the sensory evaluation of biscuits with added microencapsulated green tea extract showed that consumers’ preference for biscuits with added microencapsulated green tea extract was not significantly different from other biscuits (without or with free added green tea extract), indicating that the addition of microencapsulated green tea extract did not change consumers’ preference [93]. Recently, nano-liposomes coated with other bioactive components have been added to bread to improve its quality [95]. Therefore, it is valuable to use nanocarriers encapsulated TPs in future to improve the nutritional quality of baked foods such as bread and biscuits.

### 3.3. Active Food Packaging

Food packaging is an indispensable part of the food industry. It can effectively prevent food from being damaged by external environmental factors and extend the shelf life of food. Recently, interest in the research of intelligent and active food packaging has continued to increase. Active packaging systems are designed to “deliberately incorporate components that would release or absorb substances into or from the packaged food or the environment surrounding the food”, as defined in the European regulation (EC) No 450/2009 [96]. Thus, active packaging materials are “intended to extend the shelf-life or to maintain or improve the condition of packaged food” [96]. Among many functional active ingredients added to the packaging polymer matrix for food preservation, TPs are added because of their recognized good antioxidant and non-toxic properties [97]. However, the instability and hydrophilicity of TPs affect the stability and effectiveness of active food packaging added with TPs [98]. Therefore, the emergence of nanotechnology provides a solution to the challenge because nanomaterials have high encapsulation efficiency and slow-release characteristics [99] (Table 3).

Encapsulating TPs in nanocarriers and then adding them into polymers to make active packaging can not only improve the effectiveness of food packaging through the continuous release of TPs but also improve other properties of polymers [98,99,104,105]. Liang et al. prepared an edible chitosan film containing nanocapsules and loaded it with EGCG [99]. The nanocapsule-supplemented film exhibited better antioxidant activities, better mechanical properties and light barrier properties, which protected food from photooxidative damage [99]. Similarly, Liu et al. incorporated chitosan nanoparticles containing TPs into gelatin film. The addition of nanoparticles not only introduced antioxidant properties into the gelatin film but also increased the compactness of the film [98]. Moreover, the film achieved the sustained release of TPs in fatty food simulants [98].

With the emergence of new preparation technologies in recent years, these technologies are being applied in the preparation of nano-tea polyphenol active food packaging. Through the electrospray approach, Wang et al. prepared chitosan nanoparticles containing TPs and incorporated them into the gelatin film [36]. The composite film has good free radical scavenging ability and can extend the stability of soybean oil for more than 14 days [36]. It is a potential edible active packaging material. In addition, electrospinning technology is a simple and effective method for continuous production of nanofibers with fiber diameters ranging from sub-nanometers to micrometers [106]. It has also been used to prepare nano-tea polyphenols for active food packaging [37,101,102,103]. This technology does not require high temperatures and plasticizers; therefore, it does not lead to a loss of activity of tea polyphenol active substances that are thermally sensitive. It is a suitable approach for mixing bioactive components into polymers to produce nano composite fiber packaging materials [101]. Elsewhere, Zhang et al. prepared a cross-linked starch/tea polyphenol blend nanofiber film with enhanced mechanical, antioxidant, and hydrophobic properties through a simple one-step temperature-assisted electrospinning method [37]. The incorporation of TPs gives the film good antioxidant activity, which has potential application in active food packaging [37]. The efficacy of tea polyphenol loaded electrospun nanofiber membranes for preserving some common foods has been reported. Shao et al. used tea polyphenol-loaded pullulan-carboxymethylcellulose sodium electrospun nanofiber films to preserve strawberries [102]. During storage, the film significantly inhibited weight loss and tissue softening properties of strawberries, thereby prolonging their storage period [102]. In addition, A polyvinyl alcohol/ethyl cellulose electrospun nanofibrous film loaded with TPs has also been used in pork preservation [103]. It was found that the nanofibrous film can effectively maintain the quality of pork and prolong its shelf life due to the antioxidant and antibacterial effects of TPs [103].

## 4. Challenges and Future 

Nanotechnology is a viable approach for compensating for the poor stability of TPs. Although the technology can improve the bioavailability of TPs without destroying their biological properties, there are still challenges in the tea polyphenol nanostrategies, including the following:

(1) Most of the current research conclusions on the improvement of the stability and bioavailability of TPs by nanocarriers are mainly obtained through *in vitro* simulated environment experiments or *in vitro* cell experiments. It should be noted that the physiological environment in the human body is more complicated than these *in vitro* simulated environments. Therefore, future research should be designed on animal or clinical experimentation to verify the reliability of the described results. 

(2) To ensure the smooth development of these validation experiments, the safety of tea polyphenol nanocarriers must be guaranteed. Currently, some common tea polyphenol nanocarriers such as metal nanoparticles have low toxicity. However, the physiological toxicity of TPs nanocarriers has not been conclusively determined. On the one hand, these tea polyphenol nanocarriers are potentially toxic due to their small size, high specific surface area, and modified surface chemistry, which lead to their clearly different functional and biological properties from those exhibited by larger particles of the same material [26]. On the other hand, the dosage of these tea polyphenol nanocarriers also affects their toxicity, and high doses of nanocarriers may have higher toxicity. Therefore, future studies should pay attention to the toxicity of TP nanocarriers to ensure the development of safe and non-toxic TPs nanocarriers. 

(3) Currently, there are many studies on use of nanocarriers to enhance the targeted release of TPs in specific parts of the human body, such as the gastrointestinal tract. However, most of these carriers have not fully realized the controlled release at the target site but only reduced the release consumption of TPs in other sites to a certain extent. Therefore, how to reduce the loss of TPs in other parts and improve the targeted recognition and stable release of TPs by chemical modification/surface functionalization of nanocarrier raw materials or preparing nanocarriers with different raw materials at the same time still requires further consideration for research in the future. 

(4) At present, the preparation of TP nanocarriers is based on traditional techniques such as the ionic gelation method and the ultrasound method, etc. These techniques are complicated to operate and sometimes require harsher reaction conditions or even the introduction of other chemical substances, which undoubtedly increases the preparation cost input. In the future, researchers can apply new technologies such as electrostatic spinning and electrostatic spraying with simple operations and mild reaction conditions for the preparation of TP nanocarriers to achieve more efficient preparation. 

(5) Although TP nanocarriers have been widely used in functional ingredient delivery, functional food development, and active food packaging preparation, the applications mainly focused on the utilization of nanoparticles containing TPs. Furthermore, there are fewer applications for other nanocarriers, such as nanoemulsions, nanomicelles, nanoliposomes, and nanogels. The nanocarriers also have advantages that nanoparticles do not have, including larger loading capacities and higher absorption efficiencies. Therefore, based on advantages of TPs nanostrategies, application ranges of different TPs-nanocarriers should be expanded.

## 5. Conclusions

Tea polyphenols (TPs) are important functional components in tea with various biological activities and medicinal values. However, the applications and development of TPs are limited by their instability and low availability. With the emergence and development of tea polyphenol nano-strategies, the nanocarriers of TPs may become the “key” to break the described application barrier of TPs. At present, most of the patents on nano-sized tea polyphenols on the market focus on the development of daily necessities, bioactive materials and nutrient delivery. This paper reviewed the recent research progress in the preparation, performance, effect, and application of different types of tea polyphenol nanocarriers. On the one hand, the nanostructure can avoid the direct contact of tea polyphenols with the action site to prevent its rapid inactivation, and on the other hand, it can realize the slow and controlled release of tea polyphenols, thus improving the stability and bioavailability of tea polyphenols. Because of this, the nanocarriers have greatly promoted the application of TPs in the fields of functional ingredient delivery, functional food development, active food packaging, etc. Altogether, it is very necessary to further develop nano-structured tea polyphenols. In the future, it will be the focus of research to further realize the safe, stable, and efficient release and action of tea polyphenols through the functionalization of nanocarriers or by combining nanotechnology with other technologies.

## Figures and Tables

**Table 1:** Applications of TPs nanostrategies in functional ingredient delivery.

Nano-Carriers	Tea Polyphenols	Site of Action	Effect	Ref.
Chitosan/β-lactoglobulin nanoparticles	Epigallocatechin gallate (EGCG)	Simulated gastrointestinal conditions	The release rate and degradation of EGCG-loaded nanoparticles (20 and 60 min, respectively) in simulated stomach conditions were slower than those of the control particles (5 and 30 min, respectively)	[25]
β-lactoglobulin/gum arabic complex nanoparticles	Epigallocatechin gallate (EGCG)	Simulated gastric and intestinal fluids	The accumulative EGCG release of β-lactoglobulin/gum arabic–EGCG complex nanoparticles was only 21% after 60 min digestion in simulated gastric juice and 86% after 120 min digestion in simulated intestinal juice	[82]
Phytoferritin–chitosan–epigallocatechin nanoparticles	Epigallocatechin (EGC)	Simulated gastric/intestinal tract	The nanoparticles prepared by the heat treatment further improved the retention ratio of EGC to 38.25 ± 1.8%	[83]
Debranched starch nanoparticles	Epigallocatechin gallate (EGCG)	Simulated gastric and intestinal fluids	After 600 min, the cumulative release rate of EGCG loaded nanoparticles in simulated intestinal fluid was about 63%, and the EGCG release rate in simulated intestinal fluid was slightly higher than that in simulated gastric fluid	[62]
Nanoemulsion prepared with corn oil and polysorbate-80	Epigallocatechin gallate (EGCG)	Simulated saliva, gastric, and small intestinal fluid	The absorbed EGCG content of the nanoemulsion was significantly increased by 28.6% compared with that of TP solution	[51]
Nanoemulsion prepared with sunflower oil and Tween 80	Green tea catechins	Mimicked gastric condition	55.13 ± 1.26% of total polyphenol, 48.61 ± 0.78% of total catechins, and 46.94 ± 0.88% of EGCG were released from green tea emulsion during the first 20 h	[84]
Folic acid-functionalized nanolipid carriers	Epigallocatechin-3-gallate (EGCG)	Simulated gastric and intestinal fluids	After 3 h, the release of EGCG in simulated gastric juice was very low, about 13% and 9% of the initial amount of functional and nonfunctional nanolipid carriers. After 21 h, EGCG was released steadily in simulated intestinal fluid, with maximum cumulative release of functional and nonfunctional nanolipid carriers of 48% and 34%, respectively	[78]
Solid lipid nanoparticles	Epigallocatechin-3-gallate (EGCG)	Simulated gastric and intestinal fluids	Improved the stability of EGCG under intestinal conditions at pH 6.8	[85]

**Table 2:** Applications of TPs nanostrategies in food quality improvement.

Nano-Carriers	Tea Polyphenols	Food	Effect	Ref.
Soy lecithin liposomes	Catechin and epigallocatechin gallate	Hard low-fat cheese	Increased antioxidant properties of low-fat cheese. The treated cheese samples with liposome-encapsulated catechin or EGCG had higher FRAP (ferric reducing antioxidant power) values	[89]
Soy lecithin liposomes	Green tea catechins	Full-fat hard cheese	Increased antioxidant activity of full-fat hard cheese. The Total phenolic content (TPC), ferric reducing antioxidant power (FRAP), and oxygen radical absorbance capacity (ORAC) antioxidant activity of whole fat cheese treated with catechin after 90 days of ripening were higher than those of the control group	[90]
Nanoencapsulation	Catechin	Coconut milk	Enhanced the antioxidant activity and prolonged the shelf life of coconut milk. Coconut milk better retained antioxidant activity during 21 days of storage	[91]
Low-methoxy pectin-coated nanoliposomes	Epigallocatechin gallate	Orange juice	Maintained good stability after pasteurization and had a stronger antioxidant activity. The liposomes still showed sustained release effect for about 20 days in orange juice, and the appearance of the orange juice did not change	[92]
Protein microcapsule	Green tea catechins	Biscuits	Microcapsule can protect catechins during a thermal treatment (180 °C), but it did not improve the antioxidant capacity of biscuit dough	[93]

**Table 3:** Applications of TPs nanostrategies in active food packaging.

Nano-Carriers	Tea Polyphenols	Film Matrix	Effect	Ref.
Chitosan nanoparticles	Tea polyphenol (TP) (Catechins content ≥90%)	Gelatin films	Introduced antioxidant properties into the gelatin films and increased the compactness of films	[98]
Nanocapsule	Epigallocatechin gallate (EGCG)	Chitosan films	Increased the DPPH scavenging activity, the mechanical properties, and light barrier properties of films	[99]
Chitosan nanoparticles	Tea polyphenol (TP) (Catechins content ≥90%)	Gelatin films	Increased the antioxidant properties of films and control oil oxidation over a long term (6 weeks)	[100]
Chitosan nanoparticles	Tea polyphenol (TP)	Gelatin films	Exhibited a high ability of free radical-scavenging and prevented soybean oil oxidation for more than 14 days. Gelatin/chitosan–tea polyphenol 30% composite films showed the most delayed release of TP and had the highest DPPH radical scavenging activity of 80.50 ± 4.67% (*p* < 0.05) after 14 days	[36]
Starch nanofibers	Tea polyphenol (TP) (with a polyphenol content ≥99%)	Starch nanofibrous films	Introduced antioxidant activity into the films and increased the mechanical properties and hydrophobicity of films. The antioxidant activity of films gradually increased with increased TP content	[37]
Polylactic acid/tea polyphenol (PLA/TP) nanofibers	Tea polyphenol (TP)	Polylactic acid/tea polyphenol (PLA/TP) composite nanofibers films	Exhibited antioxidant activity and antimicrobial activities against Escherichia coli and Staphylococcus aureus. The scavenging ability of DPPH free radical was 95.07 ± 10.55% and the antibacterial activities of PLA/TP-3:1 composite fiber against Escherichia coli and Staphylococcus aureus were 92.26 ± 5.93% and 94.58 ± 6.53%, respectively	[101]
Pullulan-carboxymethylcellulose sodium-tea polyphenol (PUL-CMC-TP) nanofibers	Tea polyphenol (TP)	Pullulan-carboxymethylcellulose sodium-tea polyphenol (PUL-CMC-TP) nanofibers films	Reduced the weight loss and tissue softening properties of strawberries during storage and effectively prolonged their storage period	[102]
Poly(vinyl alcohol)/ethyl cellulose/tea polyphenol (PVA/EC-TP) nanofibers	Tea polyphenol (TP)	Poly(vinyl alcohol)/ethyl cellulose/tea polyphenol electrospun nanofibrous films	Exhibited good antioxidant and antimicrobial activity and maintain the quality of pork and prolong its shelf life by 3 days	[103]

## Data Availability

Not applicable.

## References

[1] Qu Z., Zhou S., Li P., Liu C., Yuan B., Zhang S., Liu A. (2021). Natural products and skeletal muscle health. J. Nutr. Biochem..

[2] Gong X., Ji M., Xu J., Zhang C., Li M. (2020). Hypoglycemic effects of bioactive ingredients from medicine food homology and medicinal health food species used in China. Crit. Rev. Food Sci. Nutr..

[3] Scalbert A., Johnson I.T., Saltmarsh M. (2005). Polyphenols: Antioxidants and beyond. Am. J. Clin. Nutr..

[4] Luca S.V., Macovei I., Bujor A., Miron A., Skalicka-Wozniak K., Aprotosoaie A.C., Trifan A. (2020). Bioactivity of dietary polyphenols: The role of metabolites. Crit. Rev. Food Sci. Nutr..

[5] Khan N., Mukhtar H. (2018). Tea Polyphenols in Promotion of Human Health. Nutrients.

[6] Qu Z., Liu A., Li P., Liu C., Xiao W., Huang J., Liu Z., Zhang S. (2021). Advances in physiological functions and mechanisms of (-)-epicatechin. Crit. Rev. Food Sci. Nutr..

[7] Butt M.S., Imran A., Sharif M.K., Ahmad R.S., Xiao H., Imran M., Rsool H.A. (2014). Black tea polyphenols: A mechanistic treatise. Crit. Rev. Food Sci. Nutr..

[8] Wang L., Huang X., Jing H., Ye X., Jiang C., Shao J., Ma C., Wang H. (2021). Separation of epigallocatechin gallate and epicatechin gallate from tea polyphenols by macroporous resin and crystallization. Anal. Methods.

[9] Both S., Chemat F., Strube J. (2014). Extraction of polyphenols from black tea—Conventional and ultrasound assisted extraction. Ultrason. Sonochem..

[10] Zhang T., Huang W., Jia T., Liu Y., Yao S. (2020). Ionic liquid beta-cyclodextrin-gelatin composite membrane for effective separation of tea polyphenols from green tea. Food Chem..

[11] Roh E., Kim J.E., Kwon J.Y., Park J.S., Bode A.M., Dong Z., Lee K.W. (2017). Molecular mechanisms of green tea polyphenols with protective effects against skin photoaging. Crit. Rev. Food Sci. Nutr..

[12] Qu Z., Liu A., Liu C., Tang Q., Zhan L., Xiao W., Huang J., Liu Z., Zhang S. (2021). Theaflavin Promotes Mitochondrial Abundance and Glucose Absorption in Myotubes by Activating the CaMKK2-AMPK Signal Axis via Calcium-Ion Influx. J. Agric. Food Chem..

[13] Chieng D., Kistler P.M. (2021). Coffee and tea on cardiovascular disease (CVD) prevention. Trends Cardiovasc. Med..

[14] Gan R.Y., Li H.B., Sui Z.Q., Corke H. (2018). Absorption, metabolism, anti-cancer effect and molecular targets of epigallocatechin gallate (EGCG): An updated review. Crit Rev. Food Sci. Nutr..

[15] Zhang Y., Yang H., Li S., Li W.D., Wang Y. (2021). Consumption of coffee and tea and risk of developing stroke, dementia, and poststroke dementia: A cohort study in the UK Biobank. PLoS Med..

[16] Wan J., Feng M., Pan W., Zheng X., Xie X., Hu B., Teng C., Wang Y., Liu Z., Wu J. (2021). Inhibitory Effects of Six Types of Tea on Aging and High-Fat Diet-Related Amyloid Formation Activities. Antioxidants.

[17] Teng H., Chen L. (2019). Polyphenols and bioavailability: An update. Crit. Rev. Food Sci. Nutr..

[18] Liang J., Yan H., Puligundla P., Gao X., Zhou Y., Wan X. (2017). Applications of chitosan nanoparticles to enhance absorption and bioavailability of tea polyphenols: A review. Food Hydrocoll..

[19] Zambrano-Zaragoza M.L., Quintanar-Guerrero D., González-Reza R.M., Phuong N.T., Trong O.D., Tuan A.N. (2020). Nanocontainers in food preservation: Techniques and uses. Smart Nanocontainers.

[20] Milincic D.D., Popovic D.A., Levic S.M., Kostic A.Z., Tesic Z.L., Nedovic V.A., Pesic M.B. (2019). Application of Polyphenol-Loaded Nanoparticles in Food Industry. Nanomaterials.

[21] Hu B., Liu X., Zhang C., Zeng X. (2017). Food macromolecule based nanodelivery systems for enhancing the bioavailability of polyphenols. J. Food Drug Anal..

[22] Bing H., Ting Y., Zeng X., Huang Q. (2012). Cellular uptake and cytotoxicity of chitosan–caseinophosphopeptides nanocomplexes loaded with epigallocatechin gallate. Carbohydr. Polym..

[23] Homayouni H., Kavoosi G., Nassiri S.M. (2017). Physicochemical, antioxidant and antibacterial properties of dispersion made from tapioca and gelatinized tapioca starch incorporated with carvacrol. LWT Food Sci. Technol..

[24] Zuluaga R., Guerra A.M.S., Gomez C., Velásquez-Cock J.A., Rivera N.H.C. (2019). The nanotech potential of turmeric (*Curcuma longa* L.) in food technology: A review. Crit. Rev. Food Sci. Nutr..

[25] Liang J., Yan H., Yang H.J., Kim H.W., Wan X., Lee J., Ko S. (2016). Synthesis and controlled-release properties of chitosan/β-Lactoglobulin nanoparticles as carriers for oral administration of epigallocatechin gallate. Food Sci. Biotechnol..

[26] Li Q., Liu L., Duan M., Chen X., Li J., Zhao T., Fu Y., Julian McClements D., Huang J., Lin H. (2022). TiO(2) nanoparticles negatively impact the bioavailability and antioxidant activity of tea polyphenols. Food Chem..

[27] Frias I., Neves A.R., Pinheiro M., Reis S. (2016). Design, development, and characterization of lipid nanocarriers-based epigallocatechin gallate delivery system for preventive and therapeutic supplementation. Drug Des. Devel. Ther..

[28] Dahiya S., Rani R., Kumar S., Dhingra D., Dilbaghi N. (2017). Chitosan-Gellan Gum Bipolymeric Nanohydrogels—A Potential Nanocarrier for the Delivery of Epigallocatechin Gallate. BioNanoScience.

[29] Rashidinejad A., Boostani S., Babazadeh A., Rehman A., Rezaei A., Akbari-Alavijeh S., Shaddel R., Jafari S.M. (2021). Opportunities and challenges for the nanodelivery of green tea catechins in functional foods. Food Res. Int..

[30] Ye J.-H., Augustin M.A. (2018). Nano-and micro-particles for delivery of catechins: Physical and biological performance. Crit. Rev. Food Sci. Nutr..

[31] Dai W., Ruan C., Sun Y., Gao X., Liang J. (2020). Controlled release, and antioxidant activity of chitosan and beta-lactoglobulin complex nanoparticles loaded with epigallocatechin gallate. Colloids Surf. B Biointerfaces.

[32] Sabaghi M., Hoseyni S.Z., Tavasoli S., Mozafari M.R., Katouzian I. (2021). Strategies of confining green tea catechin compounds in nano-biopolymeric matrices: A review. Colloids Surf. B Biointerfaces.

[33] Ashrafi A., Jokar M., Mohammadi Nafchi A. (2018). Preparation and characterization of biocomposite film based on chitosan and kombucha tea as active food packaging. Int. J. Biol. Macromol..

[34] Guidance D. (2014). Guidance for Industry Considering Whether an FDA-Regulated Product Involves the Application of Nanotechnology. Biotechnol. Law Rep..

[35] Ritter A. (2011). FDA Issues Draft Guidance on Nanotechnology. Biopharm. Int..

[36] Wang Y.H., Zhang R., Qin W., Dai J.W., Zhang Q., Lee K.J., Liu Y.W. (2020). Physicochemical properties of gelatin films containing tea polyphenol-loaded chitosan nanoparticles generated by electrospray. Mater. Des..

[37] Zhang D., Chen L., Cai J., Dong Q., Din Z.-u., Hu Z.-Z., Wang G.-Z., Ding W.-P., He J.-R., Cheng S.-Y. (2021). Starch/tea polyphenols nanofibrous films for food packaging application: From facile construction to enhance mechanical, antioxidant and hydrophobic properties. Food Chem..

[38] Lu Q., Li D.-C., Jiang J.-G. (2011). Preparation of a Tea Polyphenol Nanoliposome System, and Its Physicochemical Properties. J. Agric. Food Chem..

[39] Tang D.-W., Yu S.-H., Ho Y.-C., Huang B.-Q., Tsai G.-J., Hsieh H.-Y., Sung H.-W., Mi F.-L. (2013). Characterization of tea catechins-loaded nanoparticles prepared from chitosan and an edible polypeptide. Food Hydrocoll..

[40] Puligundla P., Mok C., Ko S., Liang J., Recharla N. (2017). Nanotechnological approaches to enhance the bioavailability and therapeutic efficacy of green tea polyphenols. J. Funct. Foods.

[41] Yin C., Cheng L., Zhang X., Wu Z. (2020). Nanotechnology improves delivery efficiency and bioavailability of tea polyphenols. J. Food Biochem..

[42] Yang Q.-Q., Wei X.-L., Fang Y.-P., Gan R.-Y., Wang M., Ge Y.-Y., Zhang D., Cheng L.-Z., Corke H. (2019). Nanochemoprevention with therapeutic benefits: An updated review focused on epigallocatechin gallate delivery. Crit. Rev. Food Sci. Nutr..

[43] Choi G., Kim T.-H., Oh J.-M., Choy J.-H. (2018). Emerging nanomaterials with advanced drug delivery functions; focused on methotrexate delivery. Coord. Chem. Rev..

[44] Hsieh D.-S., Wang H., Tan S.-W., Huang Y.-H., Tsai C.-Y., Yeh M.-K., Wu C.-J. (2011). The treatment of bladder cancer in a mouse model by epigallocatechin-3-gallate-gold nanoparticles. Biomaterials.

[45] Hsieh D.S., Lu H.C., Chen C.C., Wu C.J., Yeh M.K. (2012). The preparation and characterization of gold-conjugated polyphenol nanoparticles as a novel delivery system. Int. J. Nanomed..

[46] Yuan X., He Y., Zhou G., Li X., Feng A., Zheng W. (2018). Target challenging-cancer drug delivery to gastric cancer tissues with a fucose graft epigallocatechin-3-gallate-gold particles nanocomposite approach. J. Photochem. Photobiol. B Biol..

[47] Mukherjee S., Ghosh S., Das D.K., Chakraborty P., Choudhury S., Gupta P., Adhikary A., Dey S., Chattopadhyay S. (2015). Gold-conjugated green tea nanoparticles for enhanced anti-tumor activities and hepatoprotection—Synthesis, characterization and *in vitro* evaluation. J. Nutr. Biochem..

[48] David S.R., Abdullah K., Shanmugam R., Thangavelu L., Das S.K., Rajabalaya R. (2021). Green Synthesis, Characterization and In Vivo Evaluation of White Tea Silver Nanoparticles with 5-Fluorouracil on Colorectal Cancer. Bionanoscience.

[49] Barbasz A., Czyżowska A., Piergies N., Oćwieja M. (2021). Design cytotoxicity: The effect of silver nanoparticles stabilized by selected antioxidants on melanoma cells. J. Appl. Toxicol..

[50] Qin J., Liang G., Cheng D., Liu Y., Cheng X., Yang P., Wu N., Zhao Y., Wei J. (2021). Controllable synthesis of iron-polyphenol colloidal nanoparticles with composition-dependent photothermal performance. J. Colloid Interface Sci..

[51] Peng Y., Meng Q., Zhou J., Chen B., Xi J., Long P., Zhang L., Hou R. (2018). Nanoemulsion delivery system of tea polyphenols enhanced the bioavailability of catechins in rats. Food Chem..

[52] Chung J.E., Tan S., Gao S.J., Yongvongsoontorn N., Kim S.H., Lee J.H., Choi H.S., Yano H., Zhuo L., Kurisawa M. (2014). Self-assembled micellar nanocomplexes comprising green tea catechin derivatives and protein drugs for cancer therapy. Nat. Nanotechnol..

[53] Peralta-Videa J.R., Huang Y., Parsons J.G., Zhao L., Lopez-Moreno L., Hernandez-Viezcas J.A., Gardea-Torresdey J.L. (2016). Plant-based green synthesis of metallic nanoparticles: Scientific curiosity or a realistic alternative to chemical synthesis?. Nanotechnol. Environ. Eng..

[54] Ding J., Yao J., Xue J.J., Li R., Bao B., Jiang L.P., Zhu J.J., He Z.W. (2015). Tumor-Homing Cell-Penetrating Peptide Linked to Colloidal Mesoporous Silica Encapsulated (-)-Epigallocatechin-3-gallate as Drug Delivery System for Breast Cancer Therapy in Vivo. ACS Appl. Mater. Interfaces.

[55] Yu J., Yang H., Li K., Ren H., Lei J., Huang C. (2017). Development of Epigallocatechin-3-gallate-Encapsulated Nanohydroxyapatite/Mesoporous Silica for Therapeutic Management of Dentin Surface. ACS Appl. Mater. Interfaces.

[56] Yu J., Yi L., Guo R., Guo J., Huang C. (2021). The Stability of Dentin Surface Biobarrier Consisting of Mesoporous Delivery System on Dentinal Tubule Occlusion and Streptococcus Mutans Biofilm Inhibition. Int. J. Nanomed..

[57] Li X., Luo K., Lin X., Zhu C. (2019). Multifunctional Ultra-Small Nanocomplexes Capping Mesoporous Silica Nanoparticles for Multimodal Imaging and Chemo-Photothermal Therapy. ChemNanoMat.

[58] Yan C., Zhang N., Guan P., Chen P., Ding S., Hou T., Hu X., Wang J., Wang C. (2020). Drug-based magnetic imprinted nanoparticles: Enhanced lysozyme amyloid fibrils cleansing and anti-amyloid fibrils toxicity. Int. J. Biol. Macromol..

[59] Ke X., Qin N., Zhang T., Ke F., Yan X. (2019). Highly Augmented Antioxidant and Anticancer Effect of Biocompatible MIL-100(Fe)@SiO_2_-Immobilized Green Tea Catechin. J. Inorg. Organomet. Polym. Mater..

[60] Ke F., Zhang M., Qin N., Zhao G., Chu J., Wan X. (2019). Synergistic antioxidant activity and anticancer effect of green tea catechin stabilized on nanoscale cyclodextrin-based metal–organic frameworks. J. Mater. Sci..

[61] Shao G., Wang S., Zhao H., Zhao G., Yang L., Zhu L., Liu H. (2021). Tunable arrangement of hydrogel and cyclodextrin-based metal organic frameworks suitable for drug encapsulation and release. Carbohydr. Polym..

[62] Liu Q., Cai W., Zhen T., Ji N., Dai L., Xiong L., Sun Q. (2020). Preparation of debranched starch nanoparticles by ionic gelation for encapsulation of epigallocatechin gallate. Int. J. Biol. Macromol..

[63] Gou S., Chen Q., Liu Y., Zeng L., Song H., Xu Z., Kang Y., Li C., Xiao B. (2018). Green Fabrication of Ovalbumin Nanoparticles as Natural Polyphenol Carriers for Ulcerative Colitis Therapy. ACS Sustain. Chem. Eng..

[64] Bhushani J.A., Karthik P., Anandharamakrishnan C. (2016). Nanoemulsion based delivery system for improved bioaccessibility and Caco-2 cell monolayer permeability of green tea catechins. Food Hydrocoll..

[65] Chen B.-H., Hsieh C.-H., Tsai S.-Y., Wang C.-Y., Wang C.-C. (2020). Anticancer effects of epigallocatechin-3-gallate nanoemulsion on lung cancer cells through the activation of AMP-activated protein kinase signaling pathway. Sci. Rep..

[66] Chen B.H., Tsai Y.J. (2016). Preparation of catechin extracts and nanoemulsions from green tea leaf waste and their inhibition effect on prostate cancer cell PC-3. Int. J. Nanomed..

[67] Lin Y.-H., Wang C.-C., Lin Y.-H., Chen B.-H. (2021). Preparation of Catechin Nanoemulsion from Oolong Tea Leaf Waste and Its Inhibition of Prostate Cancer Cells DU-145 and Tumors in Mice. Molecules.

[68] Torchilin V.P. (2006). Micellar Nanocarriers: Pharmaceutical Perspectives. Pharm. Res..

[69] Joye I.J., Davidov-Pardo G., McClements D.J. (2014). Nanotechnology for increased micronutrient bioavailability. Trends Food Sci. Technol..

[70] Wang S., Su R., Nie S., Sun M., Zhang J., Wu D., Moustaid-Moussa N. (2014). Application of nanotechnology in improving bioavailability and bioactivity of diet-derived phytochemicals. J. Nutr. Biochem..

[71] Haratifar S., Meckling K.A., Corredig M. (2014). Antiproliferative activity of tea catechins associated with casein micelles, using HT29 colon cancer cells. J. Dairy Sci..

[72] Malekhosseini P., Alami M., Khomeiri M., Esteghlal S., Nekoei A.R., Hosseini S.M.H. (2019). Development of casein-based nanoencapsulation systems for delivery of epigallocatechin gallate and folic acid. Food Sci. Nutr..

[73] Sánchez-Giraldo V., Monsalve Y., Palacio J., Mendivil-Perez M., Sierra L., Velez-Pardo C., López B.L., Jiménez-Del-Rio M. (2020). Role of a novel (-)-epigallocatechin-3-gallate delivery system on the prevention against oxidative stress damage *in vitro* and in vivo model of Parkinson’s disease. J. Drug Deliv. Sci. Technol..

[74] Vittorio O., Curcio M., Cojoc M., Goya G.F., Hampel S., Iemma F., Dubrovska A., Cirillo G. (2017). Polyphenols delivery by polymeric materials: Challenges in cancer treatment. Drug Deliv..

[75] Chanphai P., Tajmir-Riahi H.A. (2018). Conjugation of tea catechins with chitosan nanoparticles. Food Hydrocoll..

[76] Ding J., Liang T., Min Q., Jiang L., Zhu J.-J. (2018). Stealth and Fully-Laden Drug Carriers: Self-Assembled Nanogels Encapsulated with Epigallocatechin Gallate and siRNA for Drug-Resistant Breast Cancer Therapy. ACS Appl. Mater. Interfaces.

[77] Kumar M., Tiwari A., Asdaq S.M.B., Nair A.B., Bhatt S., Shinu P., Al Mouslem A.K., Jacob S., Alamri A.S., Alsanie W.F. (2022). Itraconazole loaded nano-structured lipid carrier for topical ocular delivery: Optimization and evaluation. Saudi J. Biol. Sci..

[78] Granja A., Vieira A.C., Chaves L.L., Nunes C., Neves A.R., Pinheiro M., Reis S. (2017). Folate-targeted nanostructured lipid carriers for enhanced oral delivery of epigallocatechin-3-gallate. Food Chem..

[79] Fangueiro J.F., Calpena A.C., Clares B., Andreani T., Egea M.A., Veiga F.J., Garcia M.L., Silva A.M., Souto E.B. (2016). Biopharmaceutical evaluation of epigallocatechin gallate-loaded cationic lipid nanoparticles (EGCG-LNs): *In vivo*, *in vitro* and *ex vivo* studies. Int. J. Pharm..

[80] Barras A., Mezzetti A., Richard A., Lazzaroni S., Roux S., Melnyk P., Betbeder D., Monfilliette-Dupont N. (2009). Formulation and characterization of polyphenol-loaded lipid nanocapsules. Int. J. Pharm..

[81] Manea A.-M., Vasile B.S., Meghea A. (2014). Antioxidant and antimicrobial activities of green tea extract loaded into nanostructured lipid carriers. Comptes Rendus Chim..

[82] Gao J., Mao Y., Xiang C., Cao M., Ren G., Wang K., Ma X., Wu D., Xie H. (2021). Preparation of β-lactoglobulin/gum arabic complex nanoparticles for encapsulation and controlled release of EGCG in simulated gastrointestinal digestion model. Food Chem..

[83] Yang R., Tian J., Liu Y., Meng D., Blanchard C.L., Zhou Z. (2018). One-step fabrication of phytoferritin-chitosan-epigallocatechin shell-core nanoparticles by thermal treatment. Food Hydrocoll..

[84] Gadkari P.V., Shashidhar M.G., Balaraman M. (2017). Delivery of green tea catechins through Oil-in-Water (O/W) nanoemulsion and assessment of storage stability. J. Food Eng..

[85] Shtay R., Keppler J.K., Schrader K., Schwarz K. (2019). Encapsulation of (-)-epigallocatechin-3-gallate (EGCG) in solid lipid nanoparticles for food applications. J. Food Eng..

[86] Shpigelman A., Cohen Y., Livney Y.D. (2012). Thermally-induced β-lactoglobulin–EGCG nanovehicles: Loading, stability, sensory and digestive-release study. Food Hydrocoll..

[87] Siraj A., Naqash F., Shah M.A., Fayaz S., Majid D., Dar B.N. (2021). Nanoemulsions: Formation, stability and an account of dietary polyphenol encapsulation. Int. J. Food Sci. Technol..

[88] Piran F., Khoshkhoo Z., Hosseini S.E., Azizi M.H. (2020). Controlling the Antioxidant Activity of Green Tea Extract through Encapsulation in Chitosan-Citrate Nanogel. J. Food Qual..

[89] Rashidinejad A., Birch E.J., Sun-Waterhouse D., Everett D.W. (2016). Effect of liposomal encapsulation on the recovery and antioxidant properties of green tea catechins incorporated into a hard low-fat cheese following *in vitro* simulated gastrointestinal digestion. Food Bioprod. Processing.

[90] Rashidinejad A., Birch E.J., Everett D.W. (2016). A novel functional full-fat hard cheese containing liposomal nanoencapsulated green tea catechins: Manufacture and recovery following simulated digestion. Food Funct..

[91] Ruengdech A., Siripatrawan U. (2021). Application of catechin nanoencapsulation with enhanced antioxidant activity in high pressure processed catechin-fortified coconut milk. LWT Food Sci. Technol..

[92] Feng S.M., Sun Y.X., Wang P., Sun P.L., Ritzoulis C., Shao P. (2020). Co-encapsulation of resveratrol and epigallocatechin gallate in low methoxyl pectin-coated liposomes with great stability in orange juice. Int. J. Food Sci. Tech..

[93] Gomez-Mascaraque L.G., Hernandez-Rojas M., Tarancon P., Tenon M., Feuillere N., Ruiz J.F.V., Fiszman S., Lopez-Rubio A. (2017). Impact of microencapsulation within electrosprayed proteins on the formulation of green tea extract-enriched biscuits. LWT Food Sci. Technol..

[94] Rashidinejad A., Birch E.J., Sun-Waterhouse D., Everett D.W. (2014). Delivery of green tea catechin and epigallocatechin gallate in liposomes incorporated into low-fat hard cheese. Food Chem..

[95] Rasti B., Erfanian A., Selamat J. (2017). Novel nanoliposomal encapsulated omega-3 fatty acids and their applications in food. Food Chem..

[96] Yildirim S., Röcker B., Pettersen M.K., Nilsen-Nygaard J., Ayhan Z., Rutkaite R., Radusin T., Suminska P., Marcos B., Coma V. (2018). Active Packaging Applications for Food. Compr. Rev. Food Sci. F.

[97] Dou L., Li B., Zhang K., Chu X., Hou H. (2018). Physical properties and antioxidant activity of gelatin-sodium alginate edible films with tea polyphenols. Int. J. Biol. Macromol..

[98] Liu F., Avena-Bustillos R.J., Chiou B.-S., Li Y., Ma Y., Williams T.G., Wood D.F., McHugh T.H., Zhong F. (2017). Controlled-release of tea polyphenol from gelatin films incorporated with different ratios of free/nanoencapsulated tea polyphenols into fatty food simulants. Food Hydrocoll..

[99] Liang J., Yan H., Zhang J., Dai W., Gao X., Zhou Y., Wan X., Puligundla P. (2017). Preparation and characterization of antioxidant edible chitosan films incorporated with epigallocatechin gallate nanocapsules. Carbohydr. Polym..

[100] Liu F., Yokoyama W., Zhong F., Li Y. (2016). Preparation of gelatin films incorporated with tea polyphenol nanoparticles for enhancing controlled-release antioxidant properties. Abstr. Pap. Am. Chem. S.

[101] Liu Y.W., Liang X., Wang S.Y., Qin W., Zhang Q. (2018). Electrospun Antimicrobial Polylactic Acid/Tea Polyphenol Nanofibers for Food-Packaging Applications. Polymers.

[102] Shao P., Niu B., Chen H.J., Sun P.L. (2018). Fabrication and characterization of tea polyphenols loaded pullulan-CMC electrospun nanofiber for fruit preservation. Int. J. Biol. Macromol..

[103] Yang Y.F., Shi Y.Y., Cao X.K., Liu Q., Wang H., Kong B.H. (2021). Preparation and functional properties of poly(vinyl alcohol)/ethyl cellulose/tea polyphenol electrospun nanofibrous films for active packaging material. Food Control..

[104] Liu F., Antoniou J., Li Y., Yi J., Yokoyama W., Ma J., Zhong F. (2015). Preparation of Gelatin Films Incorporated with Tea Polyphenol Nanoparticles for Enhancing Controlled-Release Antioxidant Properties. J. Agric. Food Chem..

[105] Bao S., Xu S., Wang Z. (2009). Antioxidant activity and properties of gelatin films incorporated with tea polyphenol-loaded chitosan nanoparticles. J. Sci. Food Agric..

[106] Zhao L., Duan G., Zhang G., Yang H., He S., Jiang S. (2020). Electrospun Functional Materials toward Food Packaging Applications: A Review. Nanomaterials.

